# Compared to oxcarbazepine and carbamazepine, botulinum toxin type A is a useful therapeutic option for trigeminal neuralgia symptoms: A systematic review

**DOI:** 10.1002/cre2.882

**Published:** 2024-03-31

**Authors:** Yeganeh Naderi, Maryam Rad, Ali Sadatmoosavi, Elham Khaleghi, Zahra Khorrami, Goli Chamani, Mohammad Shabani

**Affiliations:** ^1^ Oral and Dental Diseases Research Center Kerman University of Medical Sciences Kerman Iran; ^2^ Research Center for Modeling in Health Kerman University of Medical Sciences Kerman Iran; ^3^ Ophthalmic Epidemiology Research Center, Research Institute for Ophthalmology and Vision Science Shahid Beheshti University of Medical Science Tehran Iran; ^4^ Department of Dental Medicine, Karolinska Institute Scandinavian Center for Orofacial Neuroscience (SCON) Huddinge Sweden; ^5^ Neuroscience Research Center, Neuropharmacology Institute Kerman University of Medical Sciences Kerman Iran

**Keywords:** botulinum toxin type A, carbamazepine, oxcarbazepine, trigeminal neuralgia

## Abstract

**Objectives:**

This review aimed to compare the effectiveness of three treatments: BTX A, CBZ, and OXB, in managing trigeminal neuralgia (TN).

**Material and Methods:**

We conducted a thorough search for research articles related to our issue using specific keywords on several databases, including Cochrane Central Register of Controlled Trials, Science Direct, Scopus, PubMed, Elsevier, Springer Journals, Ovid Medline, EBSCO, and Web of Science. Our focus was on publications from 1965 to 2023.

**Results:**

We retrieved 46 articles from the search and reviewed them carefully. Out of these, we selected 29 articles that met the inclusion criteria. Among the selected articles, 11 investigated the effects of CBZ and OXB, while 18 explored the impact of BTX A on the improvement of TN symptoms. The response rate ranged between 56% and 90.5% for CBZ and between 90.9% and 94% for OXB. The response rate for BTX A ranged between 51.4% and 100%. All these three treatments had a remarkable effect on the improvement of TN. Importantly, findings highlighted that side effects of CBZ and OXB could lead to treatment discontinuation in some cases, whereas BTX A's side effects have been minimal and less frequent.

**Conclusions:**

Consequently, BTX A emerges as a promising alternative for TN treatment. However, additional clinical trials are necessary to validate this finding, and further research is required to establish a standardized protocol for administering BTX A in TN.

## 1 INTRODUCTION

The hallmark of trigeminal neuralgia (TN) is abrupt, excruciating pain (Wu et al., 2012). The disease presents as short, persistent, and chronic unilateral attacks of pain in the face, usually on the right side (Merskey, 1994). This pain can last from 1 s to about 2 min (Society, 2004) and spreads along the nerve branches, especially the maxillary and mandibular branches (Chithralekha & MS, 2018). Sudden attacks of pain are triggered by stimuli that are usually not painful, such as talking, chewing, or brushing the teeth. These attacks do not threaten a person's life, but the frequency of attacks can effectively reduce a person's quality of life (Yang et al., 2018).

TN is often undiagnosed or misdiagnosed. Outbreaks have been reported in various studies having a range of 4.3–27 new cases per 100,000 people (Katusic et al., 1990; MacDonald, 2000; Mueller et al., 2011). It affects older people by a higher percentage (Shaikh et al., 2011). Women are twice as likely to be impacted as men (Katusic et al., 1990). Its lifetime prevalence in the study populations is estimated between 0.16% and 0.3% (Mueller et al., 2011; Sjaastad & Bakketeig, 2007). Ages 53 and 43, respectively, are the average onsets of classic and secondary TN, while people can develop the disease at any age, from youth to old age (De Simone et al., 2005; Maarbjerg et al., 2014). The cause of TN is still unknown. According to one of the hypotheses, the reason behind this is that the trigeminal nerve's entrance is being compressed by a cerebral artery (Katusic et al., 1990). Evidence of demilitarization and remilitarization has been provided using electron microscopy. It can also occur secondary to tumors and multiple sclerosis (De Santi & Annunziata, 2012; Nurmikko et al., 2010). According to the new classification, TN is separated into two groups: primary (idiopathic and classical) and secondary (symptomatic), depending on the underlying cause (Bendtsen et al., 2019). In acute exacerbations of attacks, intravenous injection of fosphenytoin or lidocaine can be used, and in case of long‐term treatment, carbamazepine (CBZ) or oxcarbazepine (OXB) are suggested as first‐choice medications. Lamotrigine, gabapentin, botulinum toxin type A (BTX A), pregabalin, baclofen, and phenytoin are alternative treatments. Surgical option is recommended when drug treatment is no longer effective or tolerable (Bendtsen et al., 2019).

### 1.1 Carbamazepine

Carbamazepine is a sodium channel blocker that is considered the first line of treatment for TN (Maarbjerg et al., 2017). CBZ can reduce the severity and frequency of TN attacks. However, the effectiveness of the drug introduced by the U.S. Food and Drug Administration is limited by poor tolerability (Wiffen et al., 2011). Long‐term and high‐dose use of the drug can result in side effects such as dizziness, drowsiness, leukopenia, diplopia, hyponatremia, and impaired vitamin D metabolism (Keränen & Sivenius, 1983).

### 1.2 Oxcarbazepine

OXB is a keto CBZ derivative that is quickly absorbed after oral administration. It is prescribed in many cases as the first line of classical TN treatment. Due to its high tolerance and minimal drug interactions, OXB is effective in individuals who are also taking other medications to treat their medical condition. (Jorns & Zakrzewska, 2007). The gastrointestinal tract and central nervous system are the most frequently affected areas by OXB adverse effects, which include diplopia, fatigue, drowsiness, dizziness, nausea, vomiting, and rash (Fang & Gong, 2015). Also, due to the risk of developing hyponatremia, high doses should be used with caution, and it should be noted that due to its similarity to CBZ, it can cause allergies in people allergic to CBZ (Jorns & Zakrzewska, 2007).

### 1.3 Botulinum toxin A

BTX A is a potent neurotoxin generated by the bacterium *Clostridium botulinum*, which inhibits acetylcholine from being released at neuromuscular synapses and results in paralysis of muscles (Sandrini et al., 2017). The conventional use of this toxin involves affecting muscle contraction by entering the presynaptic terminals at the neuromuscular junction. Type A botulinum toxin specifically interacts with SNAP‐25, a vital protein in acetylcholine release, while Type B binds to synaptobrevin or VAMP, preventing release into the synaptic space. However, this established mechanism does not adequately explain the pain‐relieving effects of botulinum toxin on conditions like TN, other forms of neuropathic pain, or migraines (Aurora, 2006; Castillo‐Álvarez et al., 2017).

In the treatment of TN, BTX A can be beneficial through its analgesic effects. The pain‐relieving properties of BTX A are associated with multiple mechanisms. In addition to its confirmed ability to inhibit acetylcholine exocytosis, there is evidence suggesting that BTX A may also prevent the release of different neurotransmitters, which can potentially limit muscle spindle discharge and sympathetic transmission. This impact has been associated with the inhibition of norepinephrine and ATP release, both integral to the process of chronic pain development. Furthermore, the inhibition of substance P release has been implicated, with its heightened presence in the spinal cord contributing to central sensitization—a mechanism crucial to BTX A's analgesic efficacy, particularly in addressing primary headaches (Mense, 2004; Mittal et al., 2016; Verma, 2013). Moreover, other neurotransmitters linked to the pain‐relieving effects of BTX A play a role in anti‐inflammatory mechanisms. Peptides associated with the calcitonin gene and glutamate are released together in the Gasser ganglion through calcium channel‐dependent mechanisms and implicated in migraines; in both cases, their release is inhibited by the local administration of botulinum toxin (Ashkenazi & Blumenfeld, 2013; Chan & MaassenVanDenBrink, 2014; Meng et al., 2018).

TN is regarded as one of the most agonizing pains known to mankind, which is very difficult to diagnose and treat. OXB and CBZ have been shown to have a very significant effect on the treatment of TN as the first line of treatment. On the contrary, BTX A has drawn a lot of interest in the course of therapy for this disease recently, and according to studies, it has caused few side effects. No clear results have been found on the superiority of any of these therapies over each other, nor have any studies compared the effects of BTX A with CBZ and OXB (Table 1) in clinical trials. At the same time, no systematic article was found comparing these treatments specifically. Therefore, we systematically reviewed the literature to compare them.

**Table 1:** Inclusion and exclusion criteria for the systematic review.

Inclusion criteria	Exclusion criteria
A comparison/control group or the sample included CBZ, OXB, or BTX A	CBZ, OXB, or BTX A were not included in comparison/control or sample group
Published before 2024	Duplications
Type of publication: review articles, systematic reviews, briefs, conference presentations, etc.	Dissertations and unpublished documents
Directly related to the treatment of TN	Not written in English
Available full‐text of publication	

Abbreviations: BTX A, botulinum toxin A; CBZ, carbamazepine; OXB, oxcarbazepine; TN, trigeminal neuralgia.

## 2 MATERIALS AND METHODS

### 2.1 Search strategy

This research was performed in compliance with the systematic review guidelines recommended by the Cochrane Collaboration. The PICO was used to formulate the research question (Patient/Problem, Intervention, Compared to, and Outcome) format. The subjects with TN comprised the research population. The intervention consisted of a prescription for BTX A and/or CBZ and/or OXB; the comparison (Table 2) was between individuals who received treatment with BTX A and patients treated with CBZ and/or OXB with an untreated patient cohort or control group, and the subjects' clinical status improved as a result of the study, including reduction in the intensity and frequency of paroxysmal attacks of pain. The search was undertaken across six electronic databases (i.e., Web of Science, Scopus, PubMed, Embase, Ovid, and EBSCO) systematically.

**Table 2:** Modified Jadad scores scale.

	Item	Points
Randomization	Appropriate	2
	Did not describe the details of randomization	1
	Inappropriate	0
Concealment	Appropriate	2
	Did not describe the details of concealment	1
	Inappropriate or no concealment	0
Blinded	Appropriate	2
	Did not describe the details of blinded	1
	Inappropriate or not blinded	0
Withdraw or drop‐out	Described	1
	Did not describe	0

Thesauri were used to identify the primary keywords in both singular and combined forms following the preliminary evaluations, such as medical subject headings (MeSH) and Emtree, and then the appropriate search strategy was developed based on the database. For every segment of the research question, general keywords were chosen, and synonyms were added to the search. Combinations of keywords were added to the selected subject‐specific databases, placing “AND” in between the primary keywords and “OR” between synonyms. The databases were searched from 1996 to 2023, with no time limit.

The following search phrases were used to find primary studies in different databases: (Carbamazepine OR Epitol OR Tegretol OR Finlepsin OR Neurotol OR Amizepine OR Carbamazepine Acetate OR Carbamazepine Dihydrate OR Carbamazepine Hydrochloride OR Carbamazepine l‐Tartrate (4:1) OR Carbamazepine Anhydrous OR Carbamazepine Sulfate (2:1) OR Carbamazepine Phosphate OR Carbazepin), AND (Oxcarbazepine OR Trileptal OR Timox OR GP 47680 OR N‐Formyl‐10‐oxo‐10,11‐dihydro‐5H‐dihydro[b,f]azepine‐5‐carboxamide) AND (Botulinum Toxin A OR Toxin A, Botulinum OR Botox OR Neuronox OR Oculinum OR Clostridium Botulinum Toxin Type A OR Botulinum Neurotoxin A OR Neurotoxin A, Botulinum OR OnabotulinumtoxinA OR Meditoxin OR Vistabex), AND (Trigeminal Neuralgia OR Neuralgia, Trigeminal OR Trigeminal Neuralgias OR Tic Douloureux OR Trifacial Neuralgia OR Neuralgia, Trifacial OR Trifacial Neuralgias OR Fothergill Disease OR Disease, Fothergill OR Idiopathic Trigeminal Neuralgia OR Trigeminal Neuralgia, Idiopathic OR Neuralgia, Idiopathic Trigeminal OR Idiopathic Trigeminal Neuralgias OR Secondary Trigeminal Neuralgia OR Neuralgia, Secondary Trigeminal OR Trigeminal Neuralgia, Secondary OR Secondary Trigeminal Neuralgias OR Epileptiform Neuralgia OR Epileptiform Neuralgias OR Neuralgia, Epileptiform).

To increase the sensitivity of our search, we also searched the reference lists or bibliographies of all pertinent papers and reports of current networks, pertinent organizations, and conferences.

### 2.2 Study selection

In the next step, the text documents containing the data found from the chosen databases (2654 articles in sum) were brought into EndNote X9 (Thomson Reuters) and imported into an Excel spreadsheet to look for duplicates and as a preliminary screening. Duplicates were removed. Inclusion criteria were studies reporting the effects of CBZ, OXB, and BTX A on TN. There were no restrictions on the kind of report (published, unpublished, briefs, conference presentations, etc.). Articles that were not written in English and whose data were not reachable were excluded (Table 1 shows the criteria for inclusion and exclusion). Two independent reviewers (YN and EKH) screened titles, abstracts, and main text for selection. If there was any difference of opinion among the reviewers, an arrangement was reached through consultation with the statistical advisor and the third reviewer, who is also an epidemiologist (MR). To assess the articles' quality, a numerical scale‐based method was employed. For evaluating the caliber of the randomized trials, the Jadad scale was employed and was developed for pain research. This scale presented very good validity and reliability evidence (Olivo et al., 2008). The original Jadad scale is a five‐point system. Because inadequate concealment of therapeutic allocation has been linked to an amplification of treatment effects, we decided to use a modified Jadad score scale, which provides a maximum of 2 points for concealment (Balasubramanian et al., 2006; Jiang et al., 2011). The Jadad scale score ranges from 1 to 7, which determines the quality of randomized controlled trials (RCTs). If the modified Jadad score is greater than 4, the study is considered of high quality. On the contrary, if the score is between 3 and 4, it is of moderate quality. Finally, if the score is less than 3, the study is considered of low quality. To assess the methodological quality of RCTs in a systematic review, a modified scale was used, incorporating questions as outlined in Table 2 (Chen et al., 2014).

All trials were evaluated using this checklist as a guide to identify the ones of superior quality. Articles receiving a grade greater than 3 were included in the study. To address the variability in the evaluation of recovery indicators, drug dosages, control groups, and duration of follow‐up periods in RCTs, we have incorporated retrospective studies that assessed CBZ, OXB, or BTX A as a comparison group. These studies provide valuable information, especially concerning the adverse effects of the medications, which can enhance the comprehensiveness of our research. For this purpose, the Newcastle‐Ottawa Scale (NOS) was employed to assess nonrandomized studies' quality. It is a “star system” in which a study is judged based on three perspectives: the choice of study groups, the ability to compare groups, and to determine the exposure or outcome (Wells et al., 2000).

### 2.3 Data extraction

Subsequently, the necessary data were separately extracted and added to Excel 2010 (Microsoft Corp.) through two reviewers (YN and GCh) according to a form designed to collect data, and after that, the third author assessed it (MR). Name of the principal author, publishing date, the study's quality assessment rating, type of study, sampling strategy, sample size, study group assignments, length of treatment, follow‐up duration, the drug dosage, age (average, range), gender ratio, response of the patient to treatment, kind of treatment intervention, clinical results, and complications were all methodically documented. In the occurrence of heterogeneity, we could not perform a meta‐analysis.

## 3 RESULTS

**Figure 1:**
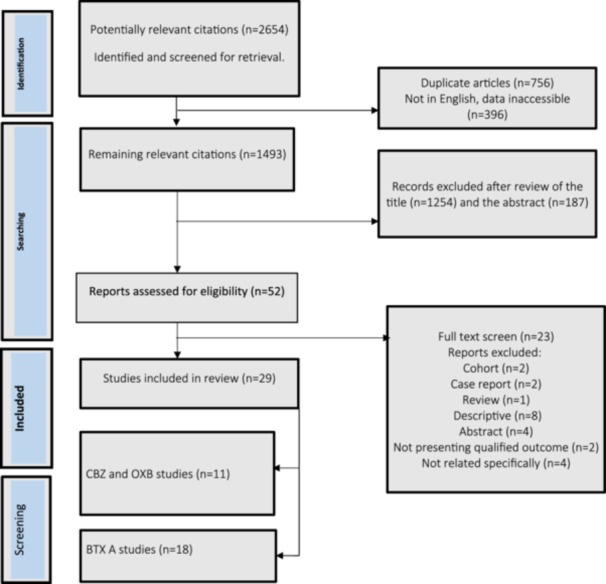
Studies considered for inclusion in the systematic review.

As indicated in Figure 1, 2654 studies were identified from the database search. Of the total records, 756 articles were eliminated after being determined to be duplicates, resulting in 1493 records. Articles not written in English and their data were not available (*n* = 396) were excluded. After excluding nonrelevant articles according to abstracts and titles, 52 full‐text articles were assessed for eligibility. Following the assessment, case reports (*n* = 2) (Bataglion et al., 2009; Zakrzewska & Patsalos, 1989), cohort studies (*n* = 2) (Caldera et al., 2018; Wu et al., 2019) and one review article (Besi et al., 2015) were not included in the research. Additionally, due to the unavailability of the full texts, four articles were eliminated (Debta et al., 2018; Hassan et al., 2013; Liebel et al., 2001; Wang & Li, 2021), eight articles were excluded due to descriptive nature (Ariyawardana et al., 2012; Ayele et al., 2020; Baykal & Kaplan, 2010; Faryabi & Joolhar, 2014; Shah et al., 2013; Taylor et al., 1981; Vilming et al., 1986; Zakrzewska et al., 2018), two articles did not show qualified outcomes (LLOYD‐SMITH & Sachdev, 1969; Nicol, 1969), and four articles did not explore what we were looking for (Ricciardi et al., 2021; Song et al., 2021; Zhang et al., 2020, 2021). Subsequently, 29 articles remained and were assessed by two authors (YN and EKH) separately.

### 3.1 CBZ and OXB effects

Among the 29 selected articles, 11 studied the effects of CBZ and OXB on the improvement of TN (Ariyawardana et al., 2003; Campbell et al., 1966; Di Stefano et al., 2014, 2021; Gomez‐Arguellese et al. 2008; Lechin et al., 1989; Lemos et al., 2010; Puri et al., 2018; Shaikh et al., 2011; Tariq et al., 2021; Verma et al., 2014; Yadav et al., 2015). A total of 1029 patients had been studied. The oldest article was related to 1966 (Campbell et al., 1966), and the latest was related to 2021 (Di Stefano et al., 2021; Tariq et al., 2021). The age range of patients was 20–84 years. The minimum sample size included 21 (Shaikh et al., 2011), and the maximum included 354 patients (Di Stefano et al., 2021). The shortest follow‐up period was 1 month (Puri et al., 2018; Tariq et al., 2021), and the longest was 87.72 months (Di Stefano et al., 2014). The lowest dose of CBZ was 100 mg (Ariyawardana et al., 2003; Tariq et al., 2021), and the highest was 1200 mg (Di Stefano et al., 2014, 2021; Lechin et al., 1989; Lemos et al., 2010; Tariq et al., 2021). The lowest dose of OXB was 300 mg (Di Stefano et al., 2021), and the maximum dose was 1800 mg (Di Stefano et al., 2014, 2021). The recovery index in several articles was a percentage of recovery (Ariyawardana et al., 2003; Campbell et al., 1966; Di Stefano et al., 2014, 2021; Gomez‐Arguelles et al., 2008; Lechin et al., 1989; Yadav et al., 2015), and in some of them was decreasing in the frequency of attacks (Campbell et al., 1966; Di Stefano et al., 2021; Gomez‐Arguelles et al., 2008; Lemos et al., 2010). The other indexes were a decrease in the degree of pain as indicated by the standard visual analog scale (VAS) (De Simone et al., 2005; Di Stefano et al., 2014; Puri et al., 2018; Tariq et al., 2021), numeric rating scale (NSR) (Lemos et al., 2010), Likert's numerical scale, and the faces rating scale developed by Wong‐Baker (Verma et al., 2014) (Tables 3 and 4 present the studies which examined the effects of CBZ and OXB).

**Table 3:** Basic information on CBZ and OXB in patients with TN is included in this systematic review.

Study design	Country	Mean age (sd)	Range age	Reference
RCT	United Kingdom	59	20–84	Campbell et al. (1966)
Double‐blind crossover trial	Venezuela	59.3	48–68	Lechin et al. (1989)
Retrospective	Sri Lanka	–	26–83	Ariyawardana et al. (2003)
Crossover (open‐label)	Spain	62.2 ± 16.7	32–84	Gomez‐Arguelles et al. (2008)
Parallel double‐blind RCT	Portugal	64 ± 12.5 68 ± 10.7		Lemos et al. (2010)
Crossover clinical trial	Malaysia	64.6 ± 13.2	32–84	Shaikh et al. (2011)
–	India	–	41–78	Verma et al. (2014)
Retrospective	Italy	67.5 ± 12.1	–	Stefano et al. (2014)
Retrospective	India	54.9	34–76	Yadav et al. (2015)
RCT	India	–	48–80	Puri et al. (2018)
Retrospective	Italy	66	29–89	Di Stefano et al. (2021)
Comparative perspective	Pakistan	54.7 ± 8.4	38–70	Tariq et al. (2021)

**Table 4:** Comparison of the effects of CBZ and OXB with control drugs in selected articles in patients with TN.

SS: (CBZ Or OXB)	SS	SS: (Comparative groups)	Follow‐up (month)	Drug dosage	Improvement criteria	Reference
36 (CBZ)	70: m = 24, f = 46	34 (placebo lactose)	2	Half a tablet four times daily, and if relief was insufficient after 48 h, it could be increased to 1 tablet four times daily.	The CBZ group improved 68% in the first period, and the placebo group only 26%.	Campbell et al. (1966)
48 (CBZ) 24 = m, 24 = f	48	48 (pimozide)	6	CBZ = 300 to 1200 mg daily, pimozide = 4 to 12 mg daily	pimozide‐treated patients' improvement was 100% and for CBZ—the treated group was 56%.	Lechin et al. (1989)
50 (CBZ)	61:26 = m, 35 = f	11 (phenytoin sodium, sodium valproate, and amitriptyline)	4	CBZ alone initial dose: 100 mg three times a day or 200 mg three times a day	62% of 50 patients initially treated with CBZ alone had significant pain control within a month; however, in the long term, treatment with CBZ alone was effective in only 29.5% of patients. The response rate of the patients treated with various combinations of CBZ and other drugs was 64% within a month.	Ariyawardana et al. (2003)
35 (OXB) 7 = m, 28 = f	35	–	3	mean maintenance dose = 773.7 mg/day	There was a significant reduction in pain frequency, leading to improvements in patient satisfaction. In general, OXB was well tolerated.	Gomez‐Arguelles et al. (2008)
24: 9 = m 15 = f	45	21 (CBZ associated with the peripheral analgesic block using ropivacaine (ROP)), 3 = m, 18 = f	5	Protocol 1: CBZ + ROP = 2 mL of a 2 mg/mL ROP and CBZ dose of 400–1000 mg/day, Protocol 2: CBZ = between 400–600 and 1000–1200 mg/day	Both protocols resulted in a decrease in pain intensity and number of pain crises. CBZ + ROP showed a significantly stronger reduction in pain intensity.	Lemos et al. (2010)
21 CBZ 9 = m, 12 = f	21	21 Lamotrigine (LTG))	–	LTG = 400 mg CBZ = 1200 mg	CBZ benefitted 90.5% (19/21) of the patients with pain relief in contrast to 62% (13/21) from LTG. VAS: 13 patients gained pain relief from LTG and 19 from CBZ, 77% (10/13) obtained a complete degree of pain relief from LTG, as compared with 21% (4/19) from CBZ.	Shaikh et al. (2011)
20 (CBZ) 12 = m, 8 = f	40	20 (duloxetine) 11 = m, 9 = f	2	Duloxetine = 20 mg CBZ = 200 mg	Duloxetine is as effective as CBZ in the treatment of TN.	Verma et al. (2014)
95 CBZ	178: 68 = m, 132 = f	83 OXB	87.72	CBZ = 600 mg (range 200–1200) OXB = 1200 mg (range 600–1800)	The initial number of responders was 98% with CBZ and 94% with OXC. In a mean period of 8.6 months, 27% of responders to CBZ discontinued treatment or dosage due to side effects. In a mean period of 13 months, the same occurred to 18% of responders to OXB.	Di Stefano et al. (2014)
69 CBZ	72: 23 = m, 49 = f	27 (CBZ and gabapentin), 21 (alcohol block) 19 (peripheral neurectomy) 5 (CBZ after surgery), 4 (referred neurologist)	36	–	CBZ: highly effective in 60.8% of the cases on a long‐term basis with maintenance doses in idiopathic TN. Other treatment modalities employed in more refractory cases include the add‐on of gabapentin, neurolytic alcohol blocks, and peripheral surgical intervention.	Yadav et al. (2015)
15 CBZ: 7 = m 8 = f	45	15 (baclofen) 5 = m,10 = f 15 (capsaicin) 6 = m,9 = f	1	CBZ = 600–800 mg/day CBZ and baclofen = 600 mg/day and baclofen 10–20 mg/day CBZ and capsaicin = 600 mg/day plus 25% capsaicin.	CBZ combined with baclofen is most effective in alleviating pain in TN when compared with CBZ alone or the CBZ capsaicin combination.	Puri et al. (2018)
179 CBZ	354: 125 = m, 229 = f	175 OXB	12	CBZ = classical, secondary and idiopathic: 600 mg (range 200–1200) OXB = classical: 1200 mg (range 300–1800) Secondary: 1200 mg (range 600–1800) Idiopathic: 900 mg (range 300–1800)	The initial proportion of responders was 88.3% with CBZ and 90.9% with OXB. In 29.6% of patients treated with carbamazepine and 12.6% of patients treated with OXB, major side effects caused discontinuation of treatment or dose reduction to an unsatisfactory level.	Stefano et al. (2021)
30 CBZ 12 = m, 18 = f	60: 21 = m, 39 = f	30 Topiramate: 9 = m, 21 = f	1	CBZ: 100–1200 mg/day Topiramate: 25–400 mg/day	Topiramate has comparable efficacy as CBZ with fewer side effects	Tariq et al. (2021)

Abbreviations: CBZ, carbamazepine; OXB, oxcarbazepine; SS, sample size; TN, trigeminal neuralgia.

### 3.2 Botulinum toxin A effect

The remaining 18 articles examined the impact of BTX A (Table 4) on TN symptom improvement (Bohluli et al., 2011; Crespi et al., 2019; Gazerani et al., 2009; Gorimanipalli et al., 2019; Jorns et al., 2019; Li et al., 2014; Liu et al., 2018; Piovesan et al., 2005; Shehata et al., 2013; Türk Börü et al., 2017; Wu et al., 2012; Xia et al., 2016; Yoshida, 2020, 2021; Zhang et al., 2014, 2017, 2019; Zuniga et al., 2008). A total of 729 patients had been examined. The oldest article was related to the year 2005 (Piovesan et al., 2005), and the latest was related to the year 2021 (Yoshida, 2021). The patients' ages ranged from 23 to 91 years. The minimum sample size included 10 (Crespi et al., 2019; Yoshida, 2020), and the maximum included 152 patients (Zhang et al., 2019). The shortest duration of follow‐up was a week (Gazerani et al., 2009), and the longest was 28 months (Zhang et al., 2019). The lowest injected dose was 20 units (Zuniga et al., 2008), and 200 units was the optimum injection dosage (Liu et al., 2018).

The recovery index in several articles was recovery percentage (Gorimanipalli et al., 2019; Li et al., 2014; Türk Börü et al., 2017; Wu et al., 2019; Zhang et al., 2014, 2017), and in some of them was a reduction in frequency of attacks (Crespi et al., 2019; Jorns et al., 2019; Lechin et al., 1989; Shehata et al., 2013; Türk Börü et al., 2017; Yoshida, 2021; Zhang et al., 2017) and several articles were decreased in the severity of the pain measured via VAS criteria (Bohluli et al., 2011; Gorimanipalli et al., 2019; Li et al., 2014; Liu et al., 2018; Piovesan et al., 2005; Shehata et al., 2013; Türk Börü et al., 2017; Wu, et al., 2012; Xia et al., 2016; Yoshida, 2020, 2021; Zhang et al., 2014, 2017, 2019; Zuniga et al., 2008). NSR was the recovery index in one article (Jorns et al., 2019). According to the articles that examined repeated injection of BTX A, no significant difference was obtained if the injection was repeated (Zhang et al., 2014; Zhang et al., 2017) (Tables 5 and 6 present the studies that examined the effects of CBZ and OXB).

**Table 5:** Basic information on patients with TN in BTX A studies is included in this systematic review.

Study design	Country	Mean age (sd)	Range age	Reference
Open‐Label Study	Brazil	F = 59.2 ± 14.2 M = 67.7 ± 6.6	–	Piovesan et al. (2005)
Open‐Label Study	Brazil	58.5	28–91	Zuniga et al. (2008)
Placebo‐Controlled Trial	Denmark	26.3 ± 2.6	23–32	Gazerani et al. (2009)
Preliminary Report	Iran	48.9	28–67	Bohluli et al. (2011)
RCT Randomized Double‐Blind Placebo‐Controlled Trial	China	59.1 ± 12.5	30–82	Wu et al. (2012)
Randomized Single‐Blinded Placebo‐Control	Egypt	45.9 ± 10	27–72	Shehata et al. (2013)
Randomized Double‐Blind Placebo‐Controlled Trial	China	58.41 ± 11.74	31–78	Zhang et al. (2014)
Open‐Label Study	China	60.6 ± 11.90	33–89	Li et al. (2014)
–	China	60.8	37–89	Xia et al. (2016)
Open‐Label Study	Turkey	54.8 ± 4.5	27–77	Türk Börü et al. (2017)
Open‐Label Trail Pilot Study	China	Single dose: 60.7 ± 10.8 Repeated dose:57 ± 10.3	–	Zhang et al. (2017)
Pilot Study	China	Older: 82.6 ± 2.9 Younger: 49.5 ± 6.3	80–90, 34–59	Liu et al. (2018)
Prospective Open‐Label Clinical Pilot Study	Thailand	67.2 + 8.4	49–79	Jorns et al. (2019)
Open‐Label Study: Pilot Study	Norway	59.4 ± 11.7	39–74	Crespi et al. (2019)
Retrospective	India	52.59 ± 12	–	Gorimanipalli et al. (2019)
Retrospective	China	F = 59, M = 60	F = 31–9, M = 39–8	Zhang et al. (2019)
Preliminary Open‐Label Uncontrolled Study	Japan	63.6 ± 13.7	40–80	Yoshida (2020)
Retrospective	Japan	68.2 ± 13.6	–	Yoshida (2021)

Abbreviations: BTX A, botulinum toxin A.; TN, trigeminal neuralgia.

**Table 6:** Comparison of the effects of BTX A with the effects of control drugs in selected articles in patients with TN.

SS: BTX.A	SS	SS: Comparative groups	Follow‐up (month)	Drug dosage	Improvement criteria	Reference
13: 4 = m, 9 = f	13		3	V1: 6.83 U V2: 6.45 U V3: 9.11 U	Pain intensity was significantly reduced at the first assessment after BTX A treatment and peaked on the 20th day.	Piovesan et al. (2005)
12: 5 = m, 7 = f	12		2	20–50 U	BTX A provides a very fast and long‐lasting benefit for the management of both TN and probably other similar conditions	Zuniga et al. (2008)
14: 14 = m	14	Placebo Saline	1 week	22.5 U	BTX A reduces pain, neurogenic inflammation, and cutaneous heat pain threshold. No alteration was recorded for electrical or pressure pain thresholds	Gazerani et al. (2009)
15: 8 = f, 7 = m	15		6	50 U	BTX A is a minimally invasive method that can play a role in treating TN before other more invasive therapies, that is, radiofrequency and surgery.	Bohluli et al. (2011)
22: 19 = m, 13 = f	42	20 (placebo) 10 = f,10 = m	3	75U/1.5 mL	Significantly more responders were present in the BTX A group (68.18%) than in the placebo group (15.00%).	Wu et al. (2012)
10	20 9 = m 11 = f	10 (placebo) Normal saline	3	40 U (8 injection points) to 60 U (12 points)	BTX A has a direct analgesic effect (significant decrease in the number of acute medications and Paroxysm frequency/day) in patients with TN.	Shehata et al. (2013)
25 (BTX‐A 25 U) 10 = m, 15 = f 28 (BTX‐A 75 U) 12 = m, 16 = f	80	27 (placebo) isotonic saline) 14 = m, 13 = f	2	BTX A 25 U = 25 U/1 mL BTX A 75 U = 75 U/1 mL	The response rates of the 25 U group (70.4%) and the 75 U group (86.2%) were significantly higher than the placebo group (32.1%) at week 8, and there was no significant difference between the 25 U and 75 U groups.	Zhang et al. (2014)
88 (BTX.A) 38 = m, 50 = f	88		14	Maximal and minimal doses were 170 U and 25 U, respectively.	Effective: within 1 month in 81 patients and at 2 months in 88 patients (100%). The therapeutic effect decreased: gradually after 3 months. The prevalence of effective treatment at 14 months: 38.6%. Complete control of pain: 22 patients (25%). No significant difference between different dose groups	Li et al. (2014)
87: 38 = m, 49 = f	87		2	Freeze‐dried BTX A 100 U/bottle with 2 ml of 0.9% sodium chloride	The effective rates after 1, 2, 4, and 8 weeks of treatment were 48.28%, 66.67%, 78.16%, and 80.46%, respectively. When compared to that before treatment, the quality of life was significantly better.	Xia et al. (2016)
27: 6 = m, 21 = f	27		6	100 U BTX A	BTX A significantly reduced pain intensity and pain attack frequency in the first week, second month, and sixth month after treatment. In the second month, 74.1% of patients, and in the sixth month, 88.9% of patients responded to treatment. Forty‐four percent of patients did not experience any pain in the sixth month.	Türk Börü et al. (2017)
44: 19 = m, 25 = f	81	37 (repeated dose): 16 = m, 21 = f	6	single dose = 70–100 U repeated dose (equal volume 2 weeks later) = 50–70 U	The response rate of the single‐dose group was 61.4% and the repeated‐dose group was 51.4%. The groups were statistically similar in TN frequency, time between treatment and effect, time to peak effect, VAS scores, and rates of adverse reactions (latency and duration). However, the single‐dose group experienced a significantly longer duration of effect.	Zhang et al. (2017)
14: ≥80 years old 4 = m,10 = f	14	29 (comparative) 60 years old< 10 = m, 19 = f	1	>80 years = 45–150 U (91.3 ± 25.6 U) <60 years = 30–200 U (71.8 ± 33.1 U)	Overall, BTX A is an effective and safe treatment for TN in patients with advanced age (≥80 years old), at dosages similar to those used in much younger counterparts (<60 years old).	Liu et al. (2018)
13	13		7	25 U/1.5 mL	Using botulinum injection with CBZ or OXB in patients with TN can reduce the severity of pain, the frequency of pain attacks, and the amount of medication taken compared to using anticonvulsant drugs alone.	Jorns et al. (2019)
10: 7 = m, 3 = f	10		3	25 IU	The reduction in the number of attacks was negative, but a significant reduction in the intensity of the attacks and pain was observed.	Crespi et al. (2019)
23: 12 = m, 11 = f	23		3	(100 IU of BTX A, Allergan) in 2.5 mL normal saline) 3 IU/cm^2^ of pain area	The response rates of both groups were 100%. The efficacy of injection was comparable in single injection and repeated injection groups.	Gorimanipalli et al. (2019)
152: 65 = m, 87 = f	152		28	Low‐dose group: <40 U Medium‐dose group: 40–70 U High‐dose group: >70 U	The overall effective rate was 89.4%. Female gender, short period of illness, and high injection dose (more than 70 units) were associated with lower long‐term VAS scores.	Zhang et al. (2019)
10: 2 = m, 8 = f	10		3	50 U	The visual analog scale score and pain frequency decreased significantly (*p* < .001). The subjective improvement achieved was 77.5% ± 13.8 % after 4 weeks, and all patients responded to treatment.	Yoshida (2020)
28: 23 = f, 5 = m	28		24.2 ± 9.1	43.1 ± 5.3 U	The mean improvement (0%, no effect; 100%, complete recovery) at the endpoint was 86.8% for TN. A significant negative correlation was observed between the number of injected sites and the improvement of the VAS at the endpoint (*p* < .005)	Yoshida (2021)

Abbreviations: BTX A, botulinum toxin A; TN, trigeminal neuralgia.

### 3.3 Side effects

The central nervous system and gastrointestinal tract were the most frequently observed CBZ adverse reactions. Nausea (Ariyawardana et al., 2003; Puri et al., 2018; Tariq et al., 2021) and vomiting (Puri et al., 2018) were the most common side effects associated with the gastrointestinal tract and dizziness (Ariyawardana et al., 2003; Di Stefano et al., 2014, 2021; Puri et al., 2018; Shaikh et al., 2011; Tariq et al., 2021), motor coordination impairment (Lechin et al., 1989), headache (Lemos et al., 2010; Puri et al., 2018), drowsiness (Ariyawardana et al., 2003; Campbell et al., 1966; Tariq et al., 2021), confusion and unbalance (Campbell et al., 1966; Di Stefano et al., 2014, 2021), muscle ataxia (Tariq et al., 2021), diplopia, lethargy, numbness of extremities (Tariq et al., 2021), fatigue, sedation (Puri et al., 2018), and somnolence (Di Stefano et al., 2014, 2021) were the most common complications associated with the central nervous system. Hepatic (Campbell et al., 1966; Di Stefano et al., 2021; Shaikh et al., 2011), renal (Shaikh et al., 2011), and hematopoietic complications, including anemia (Di Stefano et al., 2014, 2021), agranulocytosis (Campbell et al., 1966), leukopenia (Ariyawardana et al., 2003; Di Stefano et al., 2021; Di Stefano et al., 2014), and thrombocytopenia (Campbell et al., 1966; Di Stefano et al., 2021), caused by this drug have also been reported.

Inadequate secretion of vasopressin resulting in a decrease in water excretion (Ariyawardana et al., 2003), increased secretion of transaminase and gamma‐glutamyl transferase (Campbell et al., 1966; Di Stefano et al., 2021), and skin allergy reactions were also reported (Campbell et al., 1966; Di Stefano et al., 2021; Lechin et al., 1989; Shaikh et al., 2011). Considering these adverse effects on the central nervous system (often occurring together) (Di Stefano et al., 2021), hyponatremia (Di Stefano et al., 2021), leukopenia (Ariyawardana et al., 2003), and skin rash (Campbell et al., 1966) caused treatment interruption.

Side effects of OXB are reported in order of prevalence as follows: hyponatremia (Di Stefano et al., 2014, 2021; Gomez‐Arguelles et al., 2008), somnolence (Di Stefano et al., 2014, 2021; Gomez‐Arguelles et al., 2008), postural unbalance (Di Stefano et al., 2014, 2021), dizziness (Di Stefano et al., 2014, 2021; Gomez‐Arguelles et al., 2008), and complications such as liver dysfunction (Di Stefano et al., 2021), vomiting (Gomez‐Arguelles et al., 2008), nausea (Gomez‐Arguelles et al., 2008), thrombocytopenia (Di Stefano et al., 2014, 2021), headache, and muscle ataxia (Gomez‐Arguelles et al., 2008). Also, allergic skin reactions caused by OXB have been reported (Di Stefano et al., 2014, 2021). Causes of treatment interruption were hyponatremia and central nervous system side effects (Di Stefano et al., 2014, 2021; Gomez‐Arguelles et al., 2008).

The most reported complications of BTX A were conceived to be correlated to the injection approach and consisted of facial nerve palsy and facial asymmetry (Bohluli et al., 2011; Crespi et al., 2019; Gorimanipalli et al., 2019; Jorns et al., 2019; Li et al., 2014; Liu et al., 2018; Piovesan et al., 2005;  Shehata et al., 2013; Türk Börü et al., 2017; Wu et al., 2012; Yoshida, 2021; Zuniga et al., 2008), edema and swelling at the injection site (Crespi et al., 2019; Li et al., 2014; Wu et al., 2012; Xia et al., 2016; Yoshida, 2021), muscle weakness (Gazerani et al., 2009; Türk Börü et al., 2017; Xia et al., 2016; Yoshida, 2021), jaw discomfort during maximal mouth opening (Wu et al., 2012; Yoshida, 2021), tenderness, and pain at the injection site (Yoshida, 2021). Complications such as hematoma at the injection site (Shehata et al., 2013), dizziness (Jorns et al., 2019), and discomfort in the whole body (Liu et al., 2018) were also observed. Overall, the adverse reactions to BTX A were transient while slight.

### 3.4 Heterogeneity

In the event of the presence of heterogeneity in the evaluation of recovery indices, drug dose, and follow‐up time after treatment, we could not perform a meta‐analysis. Furthermore, the quantity of clinical trial studies was constrained since there were no clinical trial studies comparing botulinum toxin A with CBZ and OXB.

## 4 DISCUSSION

The review of the studies showed that CBZ, OXB, and BTX A had a remarkable effect on the improvement of TN. There are many available studies about the treatment of TN, but there are limited available systematic reviews and meta‐analysis studies to compare the treatment options of TN. A network meta‐analysis on eight drugs (including BTX A, CBZ, and OXB) reviewed related studies up to 2014. This study showed that among the drugs studied, including BTX A, lamotrigine, pimozide, tizanidine, CBZ, OXB, lidocaine, and proparacaine, all drugs except pimozide and proparacaine were more effective than placebo. CBZ, lidocaine, and BTX A showed the greatest effect on TN and can be prescribed as TN's primary stage of therapy (Yang et al., 2018).

TN does not respond to common pain relievers, and anticonvulsants are used primarily to treat TN. Through the inhibition of ectopic discharge at the disrupted membrane, a number of anticonvulsants have been reported to stabilize the plasma membrane of peripheral nerve fibers. In 70% of patients, CBZ is the most efficient anticonvulsant and is frequently prescribed for managing TN (Baykal & Kaplan, 2010). Despite being the first medication of choice for treating TN patients, CBZ has a variety of undesirable effects that may make it inappropriate for many patients. For this reason, additional medications like phenytoin, gabapentin, valproic acid, clonazepam, lamotrigine, and topiramate have been applied for the management of TN (Yoshida, 2021; Zhang et al., 2019).

OXB is a 10‐keto analog of CBZ used to treat seizures and neuropathic pain. According to certain studies, OXB is a tolerable and efficacious strategy for various types of neuropathic pain (Carrazana & Mikoshiba, 2003). It has also been shown that OXB is effective in most TN patients and is prescribed in many cases as the first treatment option (Beydoun et al., 2002). Two open‐label studies have shown that OXB notably reduces pain linked with TN, with fewer clinical complications than CBZ (Di Stefano et al., 2021; Gomez‐Arguelles et al., 2008). It is particularly effective for those who didn't respond well to CBZ previously, as it can relieve pain (Zakrzewska & Patsalos, 1989). Additionally, in a study that we were unable to systematically review due to the unavailability of the full text despite contacting the author, two drugs, CBZ and OXB, were compared and it was found that in almost all of the patients of both groups, at least a 50% reduction in the frequency of agonizing episodes occurred, and in one‐half of those enrolled in both groups, the pain was entirely controlled, which is why OXB was introduced as an effective drug with better tolerance in idiopathic TN (Liebel et al., 2001). According to a meta‐analysis comparing OXB and CBZ in patients with refractory TN, better tolerance and fewer side effects have also been reported in patients receiving OXB, and OXB was found to be an invaluable substitute therapy for TN patients (Beydoun et al., 2002).

A review of clinical trials and open trials showed that CBZ was an effective drug in TN patients' rehabilitation; nevertheless, its adverse effects were high (Campbell et al., 1966; Di Stefano et al., 2014, 2021; Gomez‐Arguelles et al., 2008; Lechin et al., 1989; Lemos et al., 2010; Puri et al., 2018; Shaikh et al., 2011). In a study comparing CBZ and OXB, the initial recovery rate was 98% for CBZ and 94% for OXB, and in an average period of 8.6 months, side effects resulted in discontinuation of treatment or dose reduction to undesirable levels in 27% of CBZ respondents. In an average of 13 months, the identical thing occurred to 18% of OXC respondents (Di Stefano et al., 2014). Additional studies also suggested that Gabapentin can be more effective (Baykal & Kaplan, 2010; Lemos et al., 2010; Puri et al., 2018). Other investigations showed that CBZ was more effective compared with pimozide (Lechin et al., 1989), Lamotrigine (Shaikh et al., 2011), and tizanidine (Vilming et al., 1986), while duloxetine and topiramate were comparable to CBZ in recovery with fewer side effects (Tariq et al., 2021; Verma et al., 2014).

BTX A is currently known as a harmless and efficient medication in TN management and has no major side effects. The effect of BTX A has been evaluated in a number of clinical trials and open‐label studies (Crespi et al., 2019; Gazerani et al., 2009; Jorns et al., 2019; Li et al., 2014; Liu et al., 2018; Shehata et al., 2013; Türk Börü et al., 2017; Wu et al., 2012; Zhang et al., 2014, 2017; Zuniga et al., 2008), showing that it is a minimally invasive treatment that can be used as a first‐line option and before other invasive treatments, such as radiation therapy and surgery, in the treatment of TN.

Additionally, Piovesan et al. showed that BTX A can also reduce preventive medication (including CBZ and OBX) by more than 50%, stop them completely, or convert multiple medications to monotherapy (Piovesan et al., 2005).

Some studies have compared single‐dose with repeated injections and, in general, showed that regarding pain relief, there was no noticeable distinction between the two groups (Zhang et al., 2014, 2017). Conversely, Zhang et al. showed that additional injections did not cause further BTX A side effects. On the other hand, patients who received moderate or high doses of BTX A had a higher complete treatment response than the low‐dose group, indicating that the injection dose was efficient in terms of treatment quality (Zhang et al., 2019).

Zhang and colleagues also discovered that there was no variance in the incidence of BTX A injection adverse effects based on gender, age, or injection dose. Nevertheless, the data demonstrated that the incidence of adverse reactions is influenced by the number of branches involved in the disease and its course; that is, individuals with moderate disease length (1–10 months) and more branches involved are more likely to have side effects (Zhang et al., 2019). Jorns and collaborators demonstrated that the usage of BTX A injection in addition to CBZ or OXB in patients with TN has the ability to lessen both the intensity and the frequency of pain attacks ( Jorns et al., 2019).

In the current study, research reviews indicated that the adverse effects of CBZ are great, and although OXB has fewer side effects than CBZ, it can still cause major side effects and may lead to discontinuation of treatment (Di Stefano et al., 2014, 2021; Gomez‐Arguelles et al., 2008). The side effects of these two medications remain a significant issue, in particular for those with idiopathic and secondary TN. It is essential to develop better‐tolerated drugs (Di Stefano et al., 2021).

Conversely, the adverse reactions to BTX‐A are mild and temporary. However, in the present study, the heterogeneity of studies performed in both groups in terms of factors such as the type of study, follow‐up period, injection dose, and, most importantly, the indicators that were evaluated for disease recovery made it impossible to perform meta‐analysis, but in general, BTX A seems to be an efficient medication with fewer adverse effects than OXB and CBZ. Sampling methods, as well as the improvement measures, were different in studies, and in some articles, they were not mentioned. Therefore, one of the limitations is that all of the articles included as part of this study lack a single definition. The inability to access the full text, and in some cases even the abstracts of related articles, is another limitation of this research.

## 5 CONCLUSION

As compared to CBZ and OXB, the study's findings suggested that BTX A is an effective medication and has fewer adverse effects, which can be recommended as a valuable substitute therapeutic option for TN management. Currently, no clinical trial has been conducted to compare the effectiveness of BTX A with CBZ and OXB. There is also no standard protocol for the administration of BTX A in TN. Thus, further clinical trials are required in the future to confirm this conclusion.

## 6 CLINICAL IMPLICATIONS

Considering that the clinical treatment of TN with CBZ and OXB has always been challenging, BTX A can help physicians manage TN as an alternative with fewer side effects.

## AUTHOR CONTRIBUTIONS

Yeganeh Naderi contributed to the study design, data collection, data interpretation, and drafting of the manuscript. Maryam Rad contributed to data collection, data analysis, and manuscript drafting. Ali Sadatmoosavi, Elham Khaleghi, and Zahra Khorrami contributed to data analysis, data interpretation, and drafting of the manuscript. Goli Chamani and Mohammad Shabani contributed to the study conception, design, data collection, data interpretation, and manuscript drafting.

## CONFLICT OF INTEREST STATEMENT

The authors declare no conflicts of interest.

## ETHICS STATEMENT

All procedures were carried out in accordance with the ethical guidelines established by the Kerman University of Medical Sciences ethics committee.

## CODE AVAILABILITY STATEMENT

This article includes all software applications that were used.

## Data Availability

Upon request, the corresponding author will provide the data sets used or analyzed in this study.
